# Shared decision making in eating disorders: the patient and clinician perspectives

**DOI:** 10.3389/fpsyt.2026.1754797

**Published:** 2026-03-11

**Authors:** Elvira Anna Carbone, Marianna Rania, Renato de Filippis, Ettore D’Onofrio, Matteo Aloi, Pasquale De Fazio, Cristina Segura-Garcia

**Affiliations:** 1 Department of Health Sciences, University Magna Græcia of Catanzaro, Catanzaro, Italy; 2 Psychiatric Unit, University Hospital Renato Dulbecco, Catanzaro, Italy; 3 Department of Clinical and Experimental Medicine, University of Messina, Messina, Italy; 4 Department of Medical and Surgical Sciences, University Magna Græcia of Catanzaro, Catanzaro, Italy

**Keywords:** anorexia nervosa, binge eating, bulimia nervosa, eating disorders, mental disorders, shared decision making, therapeutic alliance

## Abstract

**Background:**

Shared Decision Making (SDM) is essential to establish a functional and efficient patient-clinician relationship. SDM appears challenging in mental health, and even more so in eating disorders (EDs). Treatment is complex, patients’ engagement is difficult and robust evidence supporting the effectiveness of SDM in this field is still limited. This study aims to evaluate and compare SDM between patients with EDs and those with other major psychiatric conditions, and to identify potential predictors in SDM.

**Methods:**

A total of 282 outpatients with major psychiatric disorders (i.e., bipolar disorder, anxiety disorder, depressive disorder, schizophrenia spectrum disorder, EDs) were consecutively recruited. For each consultation, both the patient and the treating clinician independently completed the Shared Decision Making-Questionnaire (SDM-Q-9) and its clinician version (SDM-Q-Doc).

**Results:**

Patient ratings of SDM (SDM-Q-9) do not significantly differ across diagnostic groups, whereas clinician ratings (SDM-Q-Doc) are significantly lower for the ED group only. No differences emerge among ED diagnosis, although patients with binge eating disorder tend to have higher SDM scores. Interestingly, differences between patient and clinician views on SDM emerge at specific stages of the decision-making process. Age, education and follow-up duration significantly contribute to explaining differences in SDM from both patients’ and clinicians’ points of view.

**Conclusion:**

Patients with EDs perceive themselves as equally involved in treatment decision as those with other major mental disorders, yet clinicians report lower SDM in this group suggesting a perceptual gap. SDM in EDs is affected by socio-demographic and clinical variables. Further development of SDM techniques is crucial for improving the therapeutic alliance and addressing the cognitive and motivational features of EDs.

## 1 Introduction

The Shared Decision Making (SDM) is a dynamic exchange between clinicians and patients where both actively shape the path towards a choice aimed at supporting the patient’s well-being (1, 2).

The current definition describes clinical SDM as “*a process in which clinicians and patients work together to select tests, treatments, management or support packages, based on clinical evidence and the patient’s informed preferences; it involves the provision of evidence-based information about options, outcomes and uncertainties, together with decision support counselling and a system for recording and implementing patients’ informed preferences*” (3).

SDM is currently considered essential for informed consent (4) and patient-centered care (5–8). In recent years, SDM has been widely discussed and implemented in general medicine, whereas its role as a “positive predictive factor” in psychiatry has only recently gained recognition (9–11). Growing attention has been paid to this type of doctor-patient relationship in health care, and available evidence supports not only patient’s desire for involvement in clinical decision-making (12–20) but also a positive effects of SDM on therapeutic alliance, treatment adherence, satisfaction, trust, reduction of emotional burden, clinical outcome, hospitalization rates, and overall quality of life for people with mental illness (1, 21–28).

In this light, the importance of informing, supporting and actively involving patients regarding their disorder, potential treatments, and expected outcomes, as well as including them in the clinical decision-making process, has been emphasized (29, 30). Nevertheless, several factors can hinder the adoption of SDM model in the mental health care: limitation in mental health professionals’ communication skills or clinical experience (31), reluctance to apply SDM in an acute situation (32, 33), patient- related factors such as severity, low self-esteem, or lack of confidence in their ability to participate in decisions (21), and disease-related factors such as diagnosis, functioning or cognitive impairment (34, 35).

Although SDM has gained considerable interest and decision-support tools are available (27), its implementation in the field of eating disorders (EDs) remains particularly challenging. Several obstacles may hinder the application of SDM in this population. The ego-syntonic nature of the disorders, together with an intense fear of weight gain and the effect of malnutrition on cognitive functioning, may lead patients to underestimate the severity of their condition, resist recommended treatments, and experience difficulties in evaluating treatment options. These factors may also influence patients’ capacity to make informed treatment decisions (36). Moreover, evidence-based treatments for EDs show relatively modest remission rates (37), while approved pharmacological treatment options remain limited and strong, specific treatment recommendations are often lacking (38). Existing clinical guidelines also provide inadequate support for physicians’ critical reasoning during medication prescribing. Psychotherapeutic approaches, which are fundamental to ED treatment, further underscore the potential value of SDM as a tool to measure the level of agreement achieved and its effectiveness. Nevertheless, discussions regarding any form of treatment may provoke anxiety and discomfort in patients with EDs (38).

Collectively, these challenges highlight the need for additional evidence-based information to encourage patient inclusion, facilitate discussion and foster collaborative decision-making (39).

To date, factors influencing the implementation of Shared Decision Making have not been investigated yet in a sample of patients with Eating Disorders. Thus, this study aims to evaluate and compare SDM between patients with EDs and those with other major mental disorders, and to identify potential sociodemographic and clinical factors that may positively or negatively influence the use of SDM in routine care for the EDs population.

## 2 Materials and methods

### 2.1 Participants

Patients with EDs were recruited consecutively at the Center for Clinical Research and Treatment of Eating Disorders, University Hospital Renato Dulbecco, Italy, between September 2020 and February 2024. Age between 12 to 65 years, a diagnosis of EDs, Bipolar Disorder (BD), Major Depressive Disorder (MDD), Schizophrenia Spectrum Disorder (SSD), according to DSM-5 (40), willingness to participate, and provision of valid informed consent were the inclusion criteria. Conversely, intellective disability from mild to severe according to DSM-5 (IQ<70), neurological or medical conditions, ongoing treatment that could alter cognitive functions, substance use disorders, pregnancy, or current/recent breastfeeding within the past 12 months were considered exclusion criteria. All eligible candidates were fully informed about the aims, procedures and anonymity of the study, and a written informed consent was obtained. The study protocol was submitted and approved by the local Ethical Committee (Sezione Area Centro, Regione Calabria) before collecting any data.

### 2.2 Procedures

The diagnosis was done according to DSM-5. Experienced psychiatrists conducted clinical interviews using the Structured Clinical Interview for the DSM-5 (SCID-5-CV) (41) and the Eating Disorder Examination (EDE 17.0D) (42). The evaluation of each participant was made considering a dyad: the patient and the physician. Each patient-physician dyad rated SDM by means of two different SDM measures:

- *Shared decision making-questionnaire-clinician version* (SDM-Q-DOC). Scores range from 9 to 54, with higher scores indicating a stronger perceived therapeutic alliance (43, 44) (Cronbach’s alpha=0.700).
- *9-item shared decision making-questionnaire* (SDM-Q-9): Explores the patient’s perspective regarding the involvement and participation in the therapeutic decision-making (45, 46) (Cronbach’s alpha=0.894).
- *Symptom checklist-90-R* (SCL-90): Assesses the psychopathological symptoms (Cronbach’s alpha=0.779). In this study only the global severity index (GSI) subscale was used as it reflects overall symptoms burden and general psychological distress (47).

### 2.3 Statistical analysis

The Social Sciences Statistical Package, Version 26.0 (SPSS Inc., Chicago, IL, USA) was used for dataset management and analyses. The normality of data distribution was examined with the Shapiro-Wilk test. Descriptive statistics were computed for socio-demographic and clinical variables. The quantitative variables were expressed as mean and standard deviation (SD) and the categorical variables as frequency and percentage (%).

Group comparisons across diagnostic categories were performed using the ANCOVA for continuous variables and chi-square tests for categorical variables. *Post-hoc* pairwise comparisons were adjusted using Tukey’s correction.

Differences in SDM measures between patients and clinicians were assessed using paired-sample t-test. Effect sizes were calculated using Cohen’s d and interpreted according to conventional thresholds (0.2=small, 0.5= medium, 0.8= large).

To identify potential predictors of SDM, linear regression analyses with a forward selection method were conducted separately for the SMD-Q-9, SDM-Q-Doc and Δ%SDM (discrepancy index) as dependent variables. The Δ%SDM was calculated for each item using the formula:


$$\Delta \%SDM=\frac{\left(item_{patient}–\;item_{clinician}\right)\;x\;100}{6}$$

The divisor “6” corresponds to the maximum possible difference between the two ratings on the 0–6 Likert scale used in both SDM-Q questionnaires. This index reflects the percentage difference between patient- and clinician- reported SDM scores on a standardized 0–100 scale. Independent variables included sociodemographic and clinical factors (age, education, illness duration, follow-up, and symptoms severity). Significant coefficients were considered for p < 0.05.

## 3 Results

### 3.1 Sociodemographic and clinical characteristics

A total of 282 patients were enrolled, and their characteristics are summarized in **Table 1**. Overall, 64% of participants are female with a mean age of 41±18 years. Marital status and occupation varied considerably across diagnostic groups, with a higher proportion of single individuals and students in the ED group, whereas the other groups were more frequently married or employed. The ED group was younger and had shorter illness duration and follow-up compared with the other major mental disorder groups. Participants with BD reported the longest duration of formal education.

**Table 1:** Socio-demographics and clinic characteristics of the sample.

Variable		Overall sample		BD		MDD		SSD		AD		ED	
		Fr	%	Fr	%	Fr	%	Fr	%	Fr	%	Fr	%
Sex	Female	181	64.2	16	50	31	56.4	18	30.5	20	52.6	96	98
	Male	101	35.8	16	50	24	43.6	41	69.5	18	47.4	2	2
Age*	years	40.7	18.3	48.1	14.3	54.7	14.8	42.0	15.3	48.9	18.1	26.4	12.6
Race	Caucasian	279	98.9	32	100	54	98.2	57	96.6	38	100	98	100
	Black	1	0.3	0	0	0	0	1	1.7	0	0	0	0
	Mixed	2	0.7	0	0	1	1.8	1	1.7	0	0	0	0
Civil status	Single	153	54.2	11	34.4	11	20	41	69.5	13	34.2	77	78.6
	Married	112	39.7	18	56.2	40	72.2	15	25.4	23	60.5	16	16.3
	Divorced	10	3.5	2	6.3	1	1.8	2	3.4	1	2.6	4	4.1
	Widow/er	4	1.4	0	0	3	5.5	0	0	1	2.6	0	0
	Not available	3	1.1	1	3.1	0	0	1	1.7	0	0	1	1.0
Occupation	Unemployed	62	21.9	8	25.0	11	20.0	20	33.9	5	13.2	18	18.6
	Employed	56	19.8	7	21.9	16	29.1	10	16.9	10	26.3	13	13.4
	Housewife	25	8.9	5	15.6	6	10.9	5	8.5	7	18.4	2	2.1
	Student	73	25.9	3	9.4	4	7.3	9	15.3	4	10.5	53	54.6
	Retired	26	9.2	3	9.4	8	14.5	7	11.9	6	15.8	2	2.1
	Disabled	7	2.5	2	6.3	1	1.8	3	5.1	1	2.6	0	0
	Self employed	26	9.2	3	9.4	8	14.5	4	6.8	3	7.9	8	8.2
	Not available	7	2.4	1	3.1	1	1.8	1	1.7	2	5.3	1	1.0
Education*	years	11.4	3.6	12.4	3.4	11.2	4.2	11.9	3.4	10.4	3.8	10.4	3.3
Illness duration*	years	10.2	11.2	14.8	13.1	9.0	8.4	18.0	14.8	7.7	7.8	5.9	7.6
Follow-up*	months	39.0	50.5	52.2	50.2	58.0	66.7	47.4	53.5	40.9	47.7	18.1	27.8

*data are expressed as mean and standard deviation (SD).

AD, anxiety disorder; BD, bipolar disorder; ED, eating disorder; MDD, major depressive disorder; SSD, schizophrenia spectrum disorder.

### 3.2 Shared decision making and clinical severity

**Figure 1:**
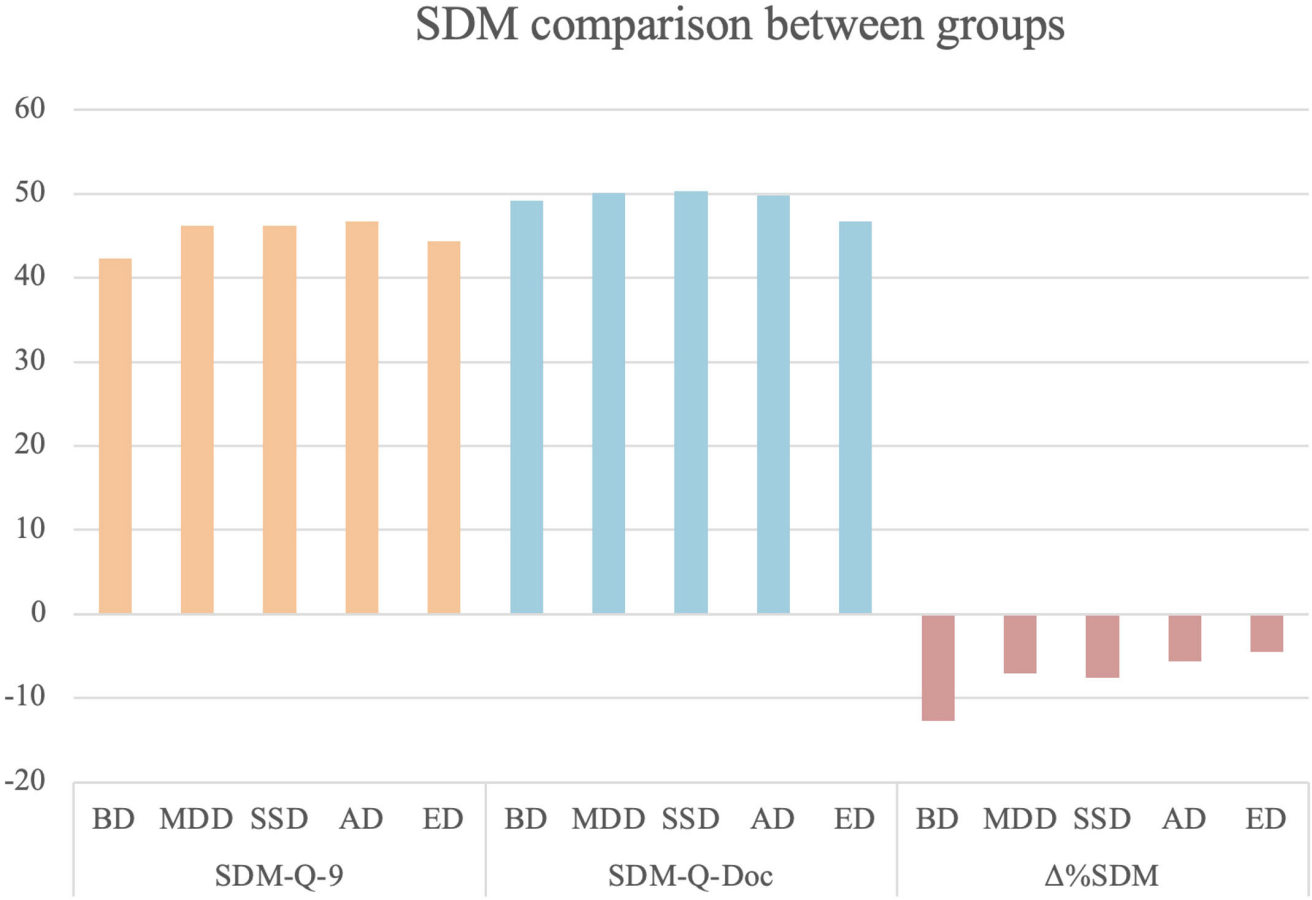
SDM comparison between groups. SDM-Q-9, Shared Decision Making-Questionnaire; SDM-Q-DOC, Shared Decision Making-Questionnaire-Doc version; Δ%SDM, delta% Shared Decision Making.

**Figure 2:**
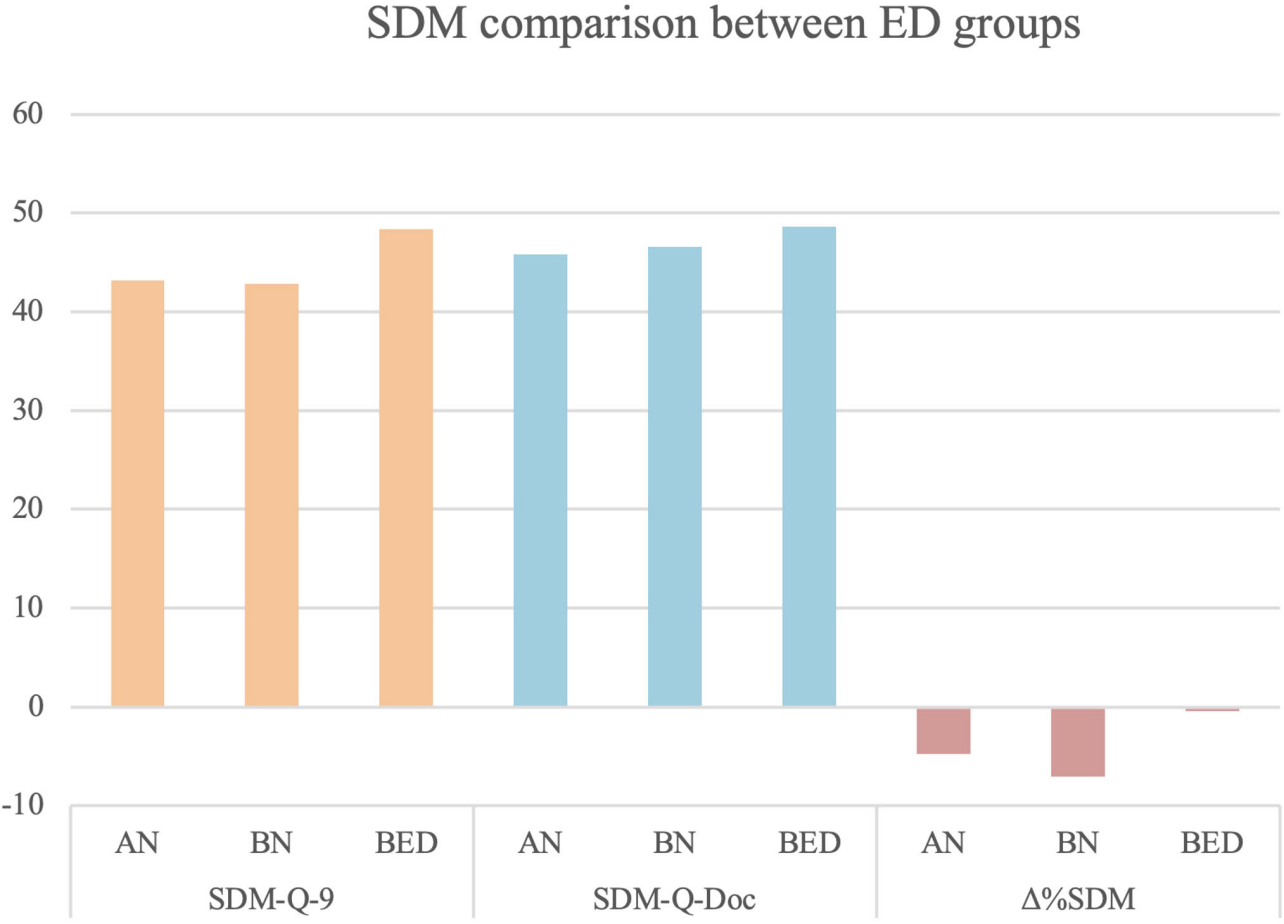
SDM comparison between ED groups. SDM-Q-9, Shared Decision Making-Questionnaire; SDM-Q-DOC, Shared Decision Making-Questionnaire-Doc version; Δ%SDM, delta% Shared Decision Making.

In the overall sample, mean SDM was 45.2±10 for patients and 48.8±5.6 for clinicians. Group comparison on SDM measures and clinical severity is presented in **Tables 2A**, **B**; **Figures 1**, **2**. No significant differences emerged among diagnostic groups in patient-reported SDM (SDM-Q-9) or in Δ%SDM. In contrast, clinician-reported SDM (SDM-Q-Doc) differed significantly across groups, with *post-hoc* analyses indicating higher scores in the MDD and SSD groups compared to the ED group. Regarding clinical severity (SCL-90 GSI), a significant effect of diagnosis was evident (p<0.001) with *post-hoc* analyses indicating lower overall symptom severity in patients with SSD compared to those with EDs.

**Table 2A:** Comparison of shared decision making and clinical severity between groups.

Variable	BD		MDD		SSD		AD		ED		F	*p*	η^2^	*Post-hoc*
	M	SD	M	SD	M	SD	M	SD	M	SD				
SDM-Q-9	42.3	11	46.2	10.4	46.2	9.1	46.7	10.3	44.4	9.7	1.390	0.237	0.020	–
SDM-Q-DOC	49.2	4.7	50.1	5.6	50.3	4.7	49.8	5.5	46.8	5.8	5.833	**<0.001**	0.078	MDD, SSD>ED
Δ%SDM	-12.7	20.3	-7.1	17.2	-7.6	16.3	-5.7	19.4	-4.5	20.2	1.242	0.293	0.018	–
SCL-90 GSI	81.6	33.9	77.5	25.3	62.7	22.1	75.8	25.3	89.9	42.6	6.463	**<0.001**	0.086	SSD<ED

AD, anxiety disorder; BD, bipolar disorder; ED, eating disorder; MDD, major depressive disorder; SSD, schizophrenia spectrum disorder; SDM-Q-9, Shared Decision Making-Questionnaire; SDM-Q-DOC, Shared Decision Making-Questionnaire-Doc version.Significant results are in bold character.

**Table 2B:** Comparison of shared decision making and clinical severity between ED groups.

Variable	AN		BN		BED	SD	F	*p*	η^2^	*Post-hoc*
	M	SD	M	SD	M					
SDM-Q-9	43.2	10.2	42.8	10.4	48.4	6.7	2.887	0.061	0.057	–
SDM-Q-DOC	45.8	6.9	46.6	5.2	48.6	4.4	1.779	0.174	0.036	–
Δ%SDM	-4.8	22.1	7.0	21.8	-0.4	13.5	0.754	0.743	0.016	–
SCL-90 GSI	87.6	26.7	92.4	29.7	90.3	72.9	0.113	0.893	0.002	–

AN, anorexia nervosa; BED, binge eating disorder; BN, bulimia nervosa; SCL-90 GSI, Symptom Check List-90 Global severity index; SDM-Q-9, Shared Decision Making-Questionnaire; SDM-Q-DOC, Shared Decision Making-Questionnaire-Doc version; Δ%SDM, delta% Shared Decision Making.

Overall, while patients’ subjective perception of SDM did not differ across diagnoses, clinicians reported lower SDM with patients with EDs compared to those with mood or psychotic disorders.

### 3.3 Shared decision making within the EDs group

Comparisons among the ED subtypes are reported in **Table 2B**. No significant differences were evident across diagnostic subgroups in patient-reported SDM, although there was a trend toward higher SDM-Q-9 scores in the Binge Eating Disorder (BED) group compared to the other subtypes. Similarly, clinician-reported SDM did not significantly differ among ED subgroups, with only a non-significant trend toward higher SDM as perceived by clinicians for patients with BED. The Δ%SDM, reflecting discrepancies between patient and clinician perceptions of SDM, showed no significant differences between groups. Finally, overall clinical severity did not differ significantly across the ED diagnostic categories.

### 3.4 Paired comparison of patient-clinician rated shared decision making in EDs

**Figure 3:**
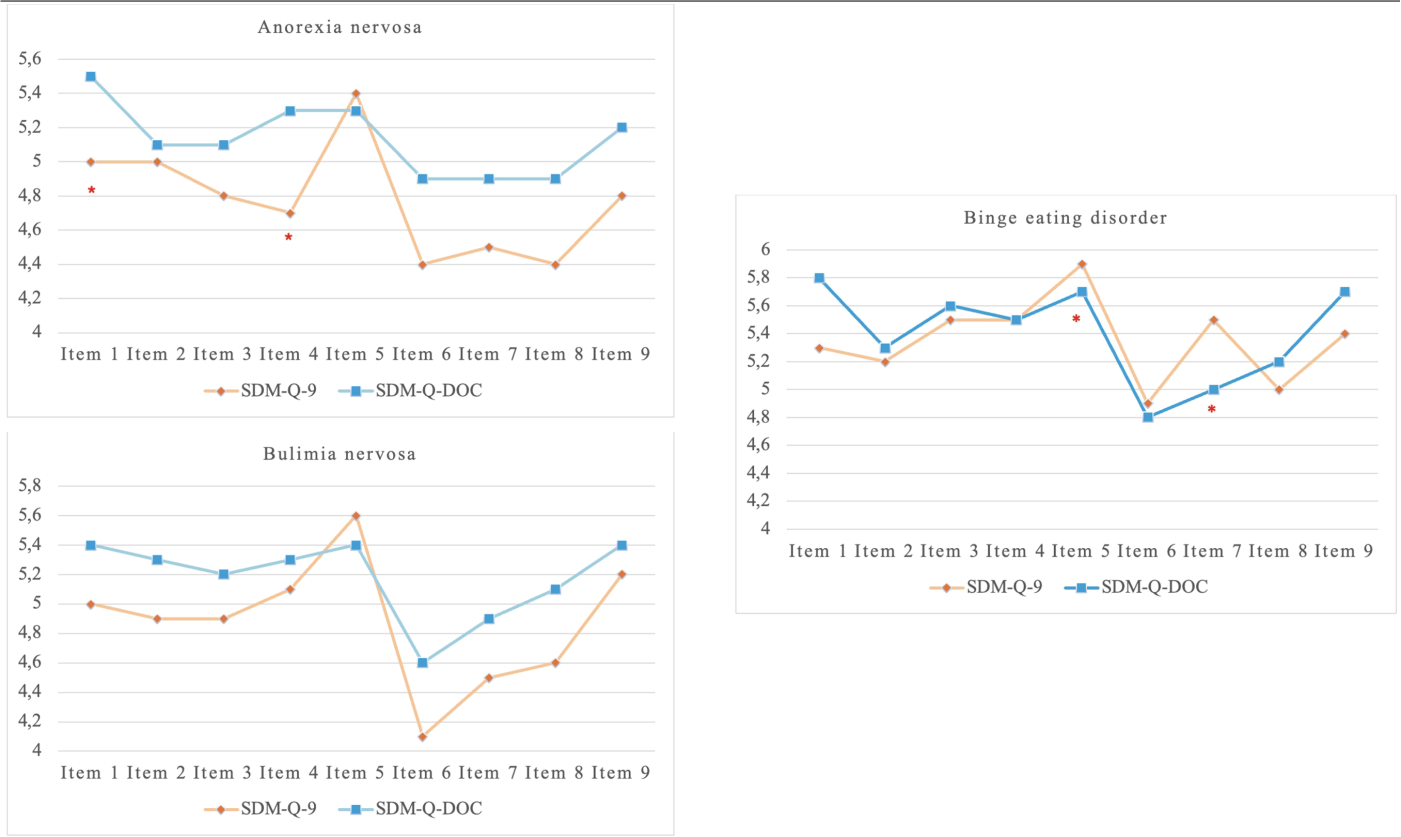
Paired sample T-test in patients with eating disorders. SDM-Q-9, Shared Decision Making-Questionnaire; SDM-Q-DOC, Shared Decision Making-Questionnaire-Doc version; Δ%SDM, delta% Shared Decision Making.

The paired sample t-test illustrates the major differences between patient- and clinician- reported SDM scores for each ED subtype (**Table 3**; **Figure 3**). Divergences were observed in perceived SDM processes between patients and clinicians, particularly in Anorexia Nervosa (AN), where clinicians reported higher engagement than patients, and in BED, where patients perceived greater shared collaboration and informational support.

**Table 3:** Paired samples T-test in patients with eating disorders.

Diagnosis	Item	SDM-Q-9		SDM-Q-DOC		t	*p*	Effect size
		M	SD	M	SD			
*AN*	Item 1	5.0	1.5	5.5	0.8	-2.152	**0.037**	-0.336
	Item 2	5.0	1.3	5.1	0.9	-0.301	0.765	-0.047
	Item 3	4.8	1.4	5.1	1.0	-1.377	0.176	-0.215
	Item 4	4.7	1.4	5.3	0.9	-2.114	**0.041**	-0.330
	Item 5	5.4	1.0	5.3	0.9	0.474	0.638	0.074
	Item 6	4.4	1.5	4.9	1.2	-1.052	0.299	-0.164
	Item 7	4.5	1.6	4.9	0.9	-1.356	0.183	-0.212
	Item 8	4.4	1.5	4.9	1.1	-1.881	0.067	-0.294
	Item 9	4.8	1.5	5.2	0.9	-1.620	0.113	-0.253
	Total score	43.2	10.2	45.8	6.9	-1.388	0.173	-0.217
*BN*	Item 1	5.0	1.5	5.4	0.7	-1.697	0.099	-0.295
	Item 2	4.9	1.6	5.3	0.6	-1.150	0.259	-0.203
	Item 3	4.9	1.3	5.2	0.7	-1.340	0.190	-0.233
	Item 4	5.1	1.3	5.3	0.8	-0.804	0.427	-0.140
	Item 5	5.6	0.7	5.4	0.6	1.314	0.198	0.229
	Item 6	4.1	1.8	4.6	0.9	-1.641	0.111	-0.290
	Item 7	4.5	1.7	4.9	0.8	-1.129	0.267	-0.196
	Item 8	4.6	1.6	5.1	0.8	-1.359	0.184	-0.240
	Item 9	5.2	1.3	5.4	0.7	-0.530	0.600	-0.094
	Total score	42.8	10.4	46.6	5.2	-1.847	0.074	-0.322
*BED*	Item 1	5.3	1.4	5.8	0.4	-1.732	0.097	-0.354
	Item 2	5.2	1.5	5.3	0.8	-0.397	0.695	-0.081
	Item 3	5.5	0.9	5.6	0.8	-0.720	0.479	-0.147
	Item 4	5.5	0.9	5.5	0.7	0.000	1.000	0.000
	Item 5	5.9	0.2	5.7	0.4	2.460	**0.022**	0.502
	Item 6	4.9	1.3	4.8	1.2	0.464	0.647	0.095
	Item 7	5.5	0.8	5.0	1.0	2.407	**0.025**	0.491
	Item 8	5.0	1.5	5.2	0.8	-0.384	0.705	-0.078
	Item 9	5.4	1.2	5.7	0.6	-0.881	0.388	-0.180
	Total score	48.4	6.7	48.6	4.4	-0.140	0.890	-0.029

AN, anorexia nervosa; BED, binge eating disorder; BN, bulimia nervosa; M, mean; SD, standard deviation.

SDM-Q-9, Shared Decision Making-Questionnaire; SDM-Q-DOC, Shared Decision Making-Questionnaire-Doc version. Effect size: Cohen’s d.Significant results are in bold character.

Interestingly, within the AN group, clinicians rated SDM significantly higher than patients on item 1 (“*My doctor made clear that a decision needs to be made”/”I made clear to my patient that a decision needs to be made*”) and item 4 (“*My doctor precisely explained the advantages and disadvantages of the treatment options”/”I precisely explained the advantages and disadvantages of the treatment options to my patient*”). In the Bulimia Nervosa (BN) group, no significant discrepancies were evident between patient and clinician ratings, although a non-significant trend suggested slightly higher clinician scores for overall SDM. In the BED group, results reveal the opposite pattern on some items. Patients rated higher SDM on item 5 (“*My doctor helped me understand all the information”/”I helped my patient understand all the information*”) and item 7 (“*My doctor and I thoroughly weighed the different treatment options”/”My patient and I thoroughly weighed the different treatment options*”).

### 3.5 Predictors of shared decision making

Forward regression analyses identified clinical and demographic predictors of SDM (**Table 4**). For patient-reported SDM (SDM-Q-9), age emerged as a positive predictor indicating that older patients reported higher perceived involvement in SDM. Conversely, follow-up duration was a negative predictor suggesting that longer clinical follow-up was associated with lower patient-related SDM. For clinician-reported SDM, education was a stronger positive predictor, indicating that clinicians perceived greater SDM with patients having higher level of education. Follow-up duration was also positively associated with SDM-Q-Doc, suggesting that longer therapeutic engagement increased clinicians’ perception of SDM. Finally, for the discrepancy index (Δ%SDM), follow-up duration negatively predicts Δ%SDM while age was a positive predictor. Accordingly, older age was associated with smaller discrepancies between patient and clinician SDM ratings, whereas longer follow-up was linked to a greater mismatch in perceived involvement.

**Table 4:** Linear regression analysis.

Dependent variable	Independent variable	B	Standard error	Beta	t	*p*	95% C.I.	
							Lower	Upper
SDM-Q-9 ^a^	Age	0.296	0.087	0.356	3.419	**<.001**	0.124	0.469
	Follow-up	-0.090	0.037	-0.250	-2.400	**0.018**	-0.164	-0.015
SDM-Q-DOC ^b^	Education	0.546	0.179	0.299	3.056	**0.003**	0.191	0.902
	Follow-up	0.043	0.021	0.199	2.036	**0.045**	0.001	0.085
Δ%SDM ^c^	Follow-up	-0.246	0.078	-0.330	-3.141	**0.002**	-0.401	-0.090
	Age	0.445	0.181	0.258	2.460	**0.016**	0.086	0.805

a Model: *R^2^ = 0.128; F = 6.694; p=0.002.* The following covariates were considered but not included: Education, Illness duration, SCL-90 GSI.

b Model: *R^2^ = 0.144; F = 7.627; p=<0.001.* The following covariates were considered but not included: Age, Illness duration, SCL-90 GSI.

c Model: *R^2^ = 0.117; F = 6.014; p=0.004.* The following covariates were considered but not included: Education, Illness duration, SCL-90 GSI.Significant results are in bold character.

## 4 Discussion

The present study examined Shared Decision Making in patients with Eating Disorders compared to those with other major psychiatric conditions and explored potential predictors of both patient- and clinician-reported SDM within the ED group. Overall, both clinicians and patients reported positive SDM experiences, indicating that SDM was effectively implemented and represented the preferred decision-making approach, in line with previous evidence (48). Our findings on patient SDM experiences were comparable to those of previous studies (49, 50), where the mean SDM ranged from 68 to 86 out of 100.

SDM scores in patients with EDs were among the lowest across diagnostic categories, yet remained comparable to those observed in bipolar disorder, major depression, schizophrenia spectrum disorders, and anxiety disorders. Within the ED sample, no significant differences emerged among AN, BN and BED, although a trend suggested slightly higher SDM levels in patients with BED and lower levels in BED. Interestingly, while patients’ subjective perceptions of SDM did not differ across diagnoses, clinicians reported significantly lower SDM with patients with EDs, particularly if compared to those with mood or psychotic disorders. This discrepancy points to a perceptual misalignment within ED dyads, particularly in specific steps of the decision-making process.

Several factors may contribute to this finding. Even though patients may feel engaged in the SDM process, this perceived involvement may not be translated into treatment adherence. Patients may understand the information provided by clinicians but unable to accept treatment options. AN and BN are highly ego-syntonic disorders and patients often fail to recognize the severity of their condition, deny illness, exhibit ambivalence toward change, inflexible thinking styles, and difficulties in evaluating risks and consequences often resulting in resistance to recommended treatment and high drop-out rates (51). Notably, these disorder-specific features, potentially intensified by the cognitive effects of malnutrition, may also affect patients’ decision-making capacity, particularly their ability to understand and evaluate treatment options within a shared decision-making framework (36). However, reduced decision-making capacity should not be assumed, as it may fluctuate across illness stages and clinical conditions. On the other hand, clinicians may exhibit mistrust and feel unable to establish a true alliance. Consistent with previous research indicating that SDM can be particularly challenging in psychiatric care (52), clinicians in our sample rated SDM as generally lower when treating patients with EDs, particularly in comparison to those with MDD and SSD. Clinicians may be reluctant to discuss potential side effects, concerned that patients might perceive them as prioritizing weight restoration or the cessation of purging behaviors over goals that patients consider more meaningful, such as anxiety reduction, improved sleep quality, and psychological support (38). This hesitation can inadvertently encourage unhelpful thinking reinforcing the ED symptoms, further complicating collaborative decision-making. Also, existing clinical practice guidelines do not consistently offer clear or positive recommendations for medication management in EDs. Instead, they often emphasize uncertainties, potential risks which may heighten clinicians’ anxiety and reduce their willing to assume full responsibility for prescribing decisions (53–55). Moreover, patients with EDs in this study reported the highest level of psychopathological severity, which may influence the level of autonomy clinicians perceive during SDM. These factors, considering that many patients are adolescents or young adults who undergo evaluation and treatment within a legal guardianship framework, may lead clinicians to perceive reduced collaboration and greater challenges in establishing a partnership, even when patients themselves feel adequately involved.

The paired patient-clinician item-level analysis sheds further light on where discrepancies arise in ED dyads. Interestingly, consistent with previous research (50), comparisons of clinician and patient SDM scores showed some divergences, demonstrating that both parties are aware of the inherent difficulties in their therapeutic relationship. Discrepancies were particularly evident in the early steps of the SDM process, such as clarifying that a decision must be made (Item 1), explaining the advantages and disadvantages of treatment options (Item 4), and jointly weighing options (Item 7). These are pivotal phases in which clinicians may strive for structure and clarity, while patients with EDs, especially those with high cognitive inflexibility or emotional distress, may perceive the interaction differently. Interestingly, patients with BED exhibited agreement with clinicians on the overall SDM score, but discrepancies emerged in specific items related to understanding information and weighing options. This suggests that, in BED, the experience of SDM may be more step-specific rather than reflecting global agreement across the entire SDM process.

This pattern may be explained by clinical differences across ED presentations: individuals with BED often report less ambivalence toward change, lower ego-syntonicity, and reduced severity of cognitive distortions, factors that may facilitate a more collaborative therapeutic attitude. From the psychiatrist’s perspective, SDM represents a substantial challenge in EDs, partly due to the limited evidence derived from randomized controlled trials. To date, pharmacological support is restricted to fluoxetine for BN and lisdexamfetamine for BED (56, 57). When treating patients who do not recognize the severity of the disease or who refuse medications due to fear of side effects, clinicians may find it difficult to balance the need to support the patient with the responsibility of explaining the possible risk associated with off-label treatments.

Regression analyses provide important insights into the demographic and clinical variables influencing SDM, underlining the significant contribution of age, education and follow-up duration. Older patients reported higher perceived involvement in SDM, potentially reflecting more mature communication skills or greater familiarity with healthcare interactions. This finding should also be interpreted in light of developmental considerations. Cognitive abilities relevant to medical decision making, including abstract reasoning, future-oriented thinking, and risk appraisal, continue to mature throughout adolescence. Younger patients may therefore demonstrate different expectations regarding participation in care and may rely more heavily on caregivers within the decision-making process (58). In contrast, older individuals often show greater autonomy and confidence in healthcare interactions, which may facilitate more active engagement in SDM. On the other hand, this may also indicate clinician’s greater propensity to involve patients with a higher functioning and less severe clinical symptomatology, consistent with Luciano et al. (35), who found greater adoption of SDM in patients with less severe illness and higher levels of interpersonal or global functioning. In contrast to that study (35), our results suggest that clinicians may be more likely to engage older patients, possibly attempting to increase adherence to treatment in the context of longer clinical histories. Conversely, longer clinical follow-up is associated with lower SDM which may suggest that over time patients become possibly critical or develop higher expectations regarding participation in SDM (59). Alternatively, it may reflect that patients are clinically stable and are not presented with therapeutic modifications that would prompt more active SDM.

For clinician-reported SDM, higher education predicted higher perceived SDM. These findings suggest that patients with greater cognitive resources and longer clinical relationships may facilitate a more collaborative SDM process. Follow-up duration also positively influenced SDM-Q-Doc scores, suggesting that longer therapeutic engagement enhances clinicians’ perception of shared involvement. Furthermore, education emerged as the strongest predictor of SDM from both clinician and patient perspective, likely reflecting the greater capacity of more highly educated patients to actively participate in SDM (60). Finally, both shorter follow-up and younger patient age were associated with larger patient-clinician discrepancies in SDM, underscoring that duration and quality of the therapeutic relationship play a central role in aligning perceptions of SDM.

Together, these findings suggest that in EDs, SDM is not inherently impaired from the patient’s perspective, but may be perceived as more challenging by clinicians due to disorder-specific interpersonal and cognitive characteristics. These results highlight the need to develop targeted strategies, techniques, and tools aimed at strengthening the patient-clinician alliance, including tailored clinician education and training in SDM approaches tailored to ED population (27). Interventions may benefit from focusing on enhancing clinicians’ skills to better navigate the unique communication patterns and motivational dynamics of patients with EDs. Practical measures may include the use of structured SDM tools, clear explanation of treatment options, psychoeducation and explicit attention during early decision-making steps. Current training programs are often considered insufficient and have a limited impact on clinicians’ knowledge and comfort with SDM (61). They could incorporate modules on caregiver engagement, communication strategies and negotiation of shared goals within the triad of patient-clinician-caregiver. Such approaches may improve alignment of expectations, reduce perceptual gaps, and foster a more effective and collaborative therapeutic alliance, ultimately supporting adherence and better clinical outcomes (28). It is noteworthy that, while SDM has been associated with improved outcomes in other mental health conditions (28), its impact on treatment outcomes specifically in patients with EDs remains to be determined. Further research is needed to explore whether enhancing SDM may improve clinical outcomes, adherence, or quality of life in this population.

The study results must be read in the light of some limitations. First, the cross‐sectional design precludes to infer about causality between predictors and SDM outcomes. Longitudinal or interventional designs are needed to establish temporal or causal relationships. Second, the ED subgroup sizes (AN, BN, BED) may have been underpowered for detecting subtle differences; the trends observed should be considered exploratory. Third, the SDM-Q-9, is a self-administered questionnaire and it may be subject to biases such as inaccurate responders, misunderstandings of items, or the tendency of patients to resent themselves more favorably or to withhold certain information, which could have influenced their answers. Furthermore, the tools used in the present study are the most validated evaluation methods in the literature to analyze the SDM from the perspective of the patient (45) and the physician (62). While the SDM measures (i.e., SDM-Q-9/SDM-Q-DOC) capture self‐reported and clinician-reported perspectives, they may not fully reflect the complexity of the decision‐making process (e.g., observed communication, nonverbal cues, or use of decision aids). Fourth, while the analyses included some key sociodemographic and clinical covariates (e.g., age, education, illness duration, follow-up, symptom severity), other potential moderators or mediators such as cognitive functioning, insight, nutritional status (particularly in EDs), therapeutic alliance, or the clinician’s training and experience with SDM principles were not assessed and may influence SDM perceptions in this population. Finally, given that the study was conducted within a single geographic/clinical setting, the generalizability of findings to other cultural contexts, healthcare systems or treatment settings (inpatient vs outpatient) may be limited.

## 5 Conclusion

In conclusion, our study provides new insights into the Shared Decision Making in Eating Disorders versus other major mental disorders. While patients with EDs report involvement levels comparable to other diagnoses, clinicians perceive lower SDM in ED care, and there are diagnosis-specific patterns of patient-clinician perception of involvement. Age, education and length of follow‐up make a modest contribution to SDM outcomes, pointing to the importance of patient characteristics and service context. These findings support the need for tailored SDM processes in ED treatment and invite further research to explore links between SDM perceptions and clinical outcomes in this vulnerable population. SDM is essential for informed consent and patient-centered care in EDs, despite the considerable challenges currently associated with its implementation including limited research, a lack of approved psychopharmacological treatment options, and insufficient guidance e on the specific pharmacological drug treatment options from clinical practice guidelines. Looking forward, further development of SDM techniques is crucial for improving the clinical and therapeutic approaches in psychiatric care, particularly for EDs. Advancing SDM strategies is essential for improving the doctor-patient relationship and promoting patient satisfaction.

## Data Availability

The datasets presented in this article are not readily available because of privacy and confidentiality restrictions. Requests to access the datasets should be directed to segura@unicz.it.
